# Prospective modeling and estimating the epidemiologically informative match rate within large foodborne pathogen genomic databases

**DOI:** 10.1186/s13104-024-06847-z

**Published:** 2024-07-09

**Authors:** Lanlan Yin, James B. Pettengill

**Affiliations:** Biostatistics and Bioinformatics Staff, Office of Analytics and Outreach, Center for Food Safety and Applied Nutrition, U. S. Food and Drug Administration, College Park, MA USA

**Keywords:** Genomics, Surveillance, Foodborne pathogen

## Abstract

**Objectives:**

Much has been written about the utility of genomic databases to public health. Within food safety these databases contain data from two types of isolates—those from patients (i.e., clinical) and those from non-clinical sources (e.g., a food manufacturing environment). A genetic match between isolates from these sources represents a signal of interest. We investigate the match rate within three large genomic databases (*Listeria monocytogenes, Escherichia coli*, and *Salmonella*) and the smaller *Cronobacter* database; the databases are part of the Pathogen Detection project at NCBI (National Center for Biotechnology Information).

**Results:**

Currently, the match rate of clinical isolates to non-clinical isolates is 33% for *L. monocytogenes*, 46% for *Salmonella*, and 7% for *E. coli*. These match rates are associated with several database features including the diversity of the organism, the database size, and the proportion of non-clinical BioSamples. Modeling match rate via logistic regression showed relatively good performance. Our prediction model illustrates the importance of populating databases with non-clinical isolates to better identify a match for clinical samples. Such information should help public health officials prioritize surveillance strategies and show the critical need to populate fledgling databases (e.g., *Cronobacter sakazakii*).

**Supplementary Information:**

The online version contains supplementary material available at 10.1186/s13104-024-06847-z.

## Objectives

With the advent of genomics and the increasing accessibility to the technology associated with it (e.g., second- and third-generation DNA sequencers), large ‘big data’ genomic databases now exist for many pathogens. These databases are ever growing because of surveillance efforts. The necessity and utility of such databases continue to be trumpeted [1]. The discussion is often focused on how to increase the size of the database via facilitating access to the technology, promoting open sharing of the data, and that equitable data use and acknowledgement practices are followed [2–4]. Often lacking from the discussion are measures of the information content of such databases and how likely they are to return actionable information regarding new samples. Some understanding of this is crucial to setting expectations and identifying gaps for improvement.

Within food safety, the NCBI Pathogen Detection Project; [5] includes large heterogenous databases for a number of pathogens such as *Salmonella enterica spp. enterica*, *Listeria monocytogenes*, and *Escherichia coli* to which whole-genome sequence (WGS) data is submitted, curated to ensure quality, and clustered. These databases are populated daily with new isolates from numerous public health agencies, academic institutions, and others throughout the world (e.g., [6]). For public health, these databases are routinely surveilled to detect signals of interest such as recent clinical isolates matching food or environmental isolates; this match between WGS data from different isolates generates the hypothesis that the source of the food or environmental isolates is the source of human illness. Such a hypothesis may then be confirmed via additional data sources and follow-up including epidemiological and traceback [7].

Here, we investigated the characteristics of large genomic databases of *E. coli, Salmonella,* and *L. monocytogenes* and the relatively small *Cronobacter spp.* database that support food safety and public health. Our primary objective is to explore the behavior of match rate over time and determine whether we can forecast and predict the match rate under certain circumstances. In doing so, we investigated the characteristics of the database, such as database size, the proportion represented by non-clinical isolates and the inherent genetic diversity of the pathogen. We also evaluated a logistic regression model to predict future database behavior.

## Data description

### Data collection and calculation of match rate

We investigated four genomic databases (*L. monocytogenes*, *Escherichia coli*, *Salmonella*, and *Cronobacter spp.*) that are part of NCBI’s Pathogen Detection project; the data analyzed here were downloaded on Feb 28, 2023 (Table 1). Based on the epi_type metadata attribute, BioSamples were assigned as either “clinical” or “environmental” (“environmental” also includes isolates from products and other non-clinical sources). Data with epi_type NULL were excluded from the analyses. For each of the taxonomic groups, historical datasets were created for each quarter by including all clinical BioSamples with target_creation_date in that period and all environmental BioSamples with target_creation_date within and before that period; target_creation_date is a metadata attribute that represents the date that an isolate’s WGS data showed up in the Pathogen Detection database. It is important to note that the genomic data in the database are from numerous global public health agencies, academic institutions, and other groups throughout the world. They are not a random sample of the pathogens present in the built and natural environment or found within the food products; the clinical data are predominantly from patients who visited a clinic as a result of being infected with a foodborne pathogen.

**Table 1:** Number of BioSamples per taxon, source (clinical or env.) and the match rate in the database at the end of year 2022

Species	NCBI’s PDG#	Total number of Biosamples	Clinical Biosamples^a^	Env. Biosamples^a^	Clinical Matches^b^
*Cronobacter*	PDG000000043.171	1140	221 (19%)	825 (72%)	31 (14%)
*E. coli*	PDG000000004.3646	285,547	158,717 (56%)	44,714 (16%)	11,243 (7%)
*Listeria*	PDG000000001.3127	54,555	17,640 (32%)	30,803 (56%)	5812 (33%)
*Salmonella*	PDG000000002.2581	506,936	351,287 (69%)	119,791 (24%)	161,729 (46%)

^a^Numbers in parentheses denote percentage of all BioSamples for the specified taxon

^b^Numbers in parentheses denote the percentage of clinical BioSamples that find matched non-clinical BioSamples in the database

For estimates of the pairwise SNP distance among isolates to determine whether two isolates match or not, we used the delta_positions_unambiguous, the number of positions where two isolates have different states and those states are unambiguous, within the SNP_distance.tsv for each pathogen provided by the Pathogen Detection Project (see https://ftp.ncbi.nlm.nih.gov/pathogen/ReadMe.txt for more information). We used a SNP distance threshold of 20 to determine whether any clinical BioSamples were a match to environmental biosamples; 20 is a general SNP distance threshold used in the interpretation of WGS from foodborne pathogens [7]. We note that in practice a single threshold may not be appropriate where because of taxon specific differences in genetic diversity and evolutionary dynamics (e.g., [8]) a more customized threshold could be used. The match rate of clinical samples to environmental samples in each quarter period was computed as the ratio of the number of matches to the total number of clinical BioSamples.

### Match rate variability

**Fig. 1:**
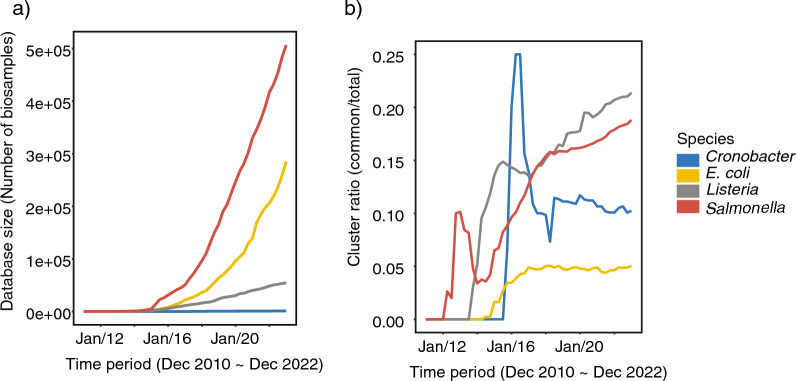
**a** The growth of sequence data for four foodborne pathogens within NCBI’s pathogen detection database. **b** Fraction of the total number of clusters that are “common” clusters (i.e., those containing both clinical and environmental BioSamples)

**Fig. 2:**
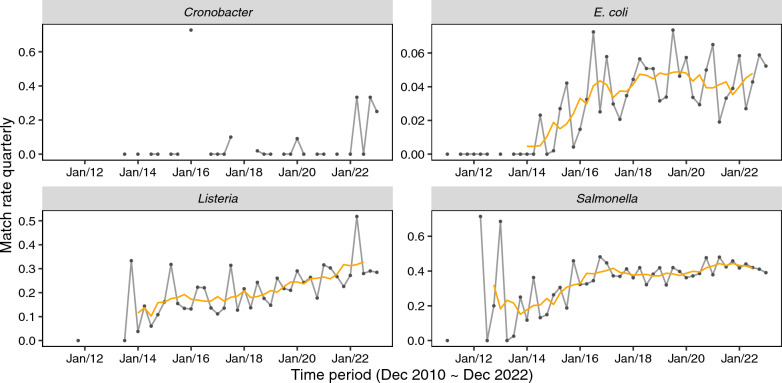
Fluctuations in match over time for four species. A simple moving average (taking average of previous 2 data points, current data point, and next 2 data points) curve in orange was added for *Salmonella, E. coli, and Listeria* to accentuate the variation of match rate over the years. Note differences in the scale of the y-axis

In the past decade, the numbers of BioSamples in the NCBI Pathogen Detection database has grown rapidly for Salmonella, *E*. *coli*, and *L*. *monocytogenes* (Fig. 1a). At the end of 2022 (data before then constitutes what was analyzed here), *Salmonella* was the largest database (N = 506,936) followed by *E. coli* (N = 285,547) and *L. monocytogenes* (N = 54,555) (Table 1). Noticeably, there are only 1,140 Cronobacter spp. BioSamples in the database with more than half of the records created after 2018. As the database size growth rate increased rapidly from year 2014 to 2018 there was a corresponding increase in the match rate of each species (Fig. 2). This may be an artifact of how various public health agencies populated the database where, perhaps, a large collection of clinical isolates was deposited, and non-clinical isolates followed and gained pace of submission. Taking all BioSamples deposited in the database before Dec 31, 2022 into account, 46% *Salmonella* clinical BioSamples and 33% *L. monocytogenes* BioSamples matched non-clinical BioSamples, and surprisingly *E. coli*, with the second largest database size, only has a match rate of 7%.

We found that there were drastic fluctuations in match rates, except for *Cronobacter spp.,* during the primary stage when the databases were small (Fig. 2; see Supplemental Table S1 for more information). This was especially pronounced when the database size was less than 1000 samples. With still only 1140 *Cronobacter spp.* BioSamples in the database, it is not surprising that the match rate has varied greatly since it was created over 10 years ago.

### Genetic diversity

To help explain why there are differences in the match rate among the taxa, we explored the number of total clusters in each database and the number of clusters that contain both environmental and clinical isolates (i.e., heterogenous sources) (Fig. 1b). The percent of clusters with isolates from heterogenous sources for *Salmonella* (19%) and *L. monocytogenes* (21%) look to still be increasing (Fig. 1b). In contrast, only about 5% of *E. coli* clusters contain both clinical and environmental isolates despite it having about three times as many clinical to non-clinical samples in the database that is similar to Salmonella (*L. monocytogenes* is the opposite and has 1.6 as many non-clinical samples to clinical samples)*.* This suggests that for *E. coli* either the putative source of clinical samples has not been sampled or the non-clinical isolates have not contributed to illness. Another potential contributing factor to the low match rate for *E. coli* is that there are clinical isolates with an isolation source of “urine” or similar suggesting that they are not the result of foodborne pathogens but rather urinary tract infections. However, those isolates are only 6.7% of the clinical isolates. Also of note with respect to *E. coli* is that we did not consider pathogenicity differences among *E. coli* in analyzing the data, which is complex but incorporating such information (i.e., virulence) in future work may also explain in part the low match rate. Additionally, *E. coli* does seem to have a higher genetic diversity and more genetic substructure than the other taxa investigated making matches less likely.

### Modeling and prediction of match rate

First, simple logistic regressions were applied to explore relationship between quarterly match rate and seven database feature variables respectively in the following form:

$$\text{log}\left(\frac{p}{1-p}\right)={\beta }_{0}+{\beta }_{1}{x}_{1}$$

where $p$ is the probability of clinical match, ${x}_{1}$ is a predictor variable (one of the database features), ${\beta }_{0}$ and ${\beta }_{1}$ are the regression coefficients. A positive ${\beta }_{1}$ implies that increasing ${x}_{1}$ is associated with higher $p$. The fitted models were evaluated by the Akaike information criterion (AIC). Lower AIC and RSE suggest better fitting. In addition, pseudo-R square by McFadden was calculated as an indicative of improvement from the null model to the current model.

Seven database features we studied are: database size (number of total BioSamples in database), number of environmental biosamples, number of clinical biosamples, number of heterogenous clusters (those that contain both environmental and clinical BioSamples), percentage of environmental biosamples, percentage of clinical biosamples, and cluster ratio(heterogenous clusters/total). Quarterly match rates were calculated for observations ending on December 31, 2021, and data with database size less than 1000 were excluded from model fitting due to instability. All variables were significantly related to match rate (p < 0.001). Cluster ratio ranks highest with the lowest AIC values and highest McFadden’s R squared (Table 2). An increase of 0.5% in heterogeneity is associated with an increase of 10% in the odds of getting matched clinical BioSamples.

**Table 2:** Logistic regression and the variables related to match rate

Variable	AIC	Coefficient*	Std. Error	P val	McFadden’s R^2^
Cluster ratio(heterogeneous/total)	12,354	1.89E + 01 (0.5%)	9.67E−02	< 0.001	0.820
Number of total environmental BioSamples	32,499	2.18E−05 (4377)	1.20E−07	< 0.001	0.527
Number of heterogeneous clusters	33,399	5.54E−04 (172)	3.07E−06	< 0.001	0.514
Number of total BioSamples	48,450	4.01E−06 (23,748)	2.87E−08	< 0.001	0.295
Number of total clinical samples	49,417	5.53E−06 (17,219)	4.03E−08	< 0.001	0.281
Percentage of environmental BioSamples	58,218	2.95E + 00 (3%)	2.87E−02	< 0.001	0.153
Percentage of clinical BioSamples	68,634	− 3.88E−01 (25%)	3.57E−02	< 0.001	0.002

The numbers in parentheses show the change in the predictor variable that is required to increase odds of getting matches by 10%

In the case of a negative coefficient, it is the reduction needed in the predictor variable to increase odds of getting matches by 10%

After identifying match rate related variables through logistic regression, we built multiple logistic regression models with all pairwise possible combinations of variables in the following form:

$$\text{log}\left(\frac{p}{1-p}\right)={\beta }_{0}+{\beta }_{1}{x}_{1}+{\beta }_{2}{x}_{2}+{\beta }_{3}{x}_{1}{x}_{2}$$

where $p$, ${\beta }_{0}$, ${\beta }_{1}$, and ${x}_{1}$ have the similar meaning in simple logistic regression, ${x}_{2}$ is the second predictor variable, ${\beta }_{2}$ and ${\beta }_{3}$ are other two regression coefficients. If coefficient (${\beta }_{3})$ of an interaction term ${x}_{1}{x}_{2}$ is significant, it indicates that the effect of ${x}_{1}$ on $p$ depends on ${x}_{2}$.

The best fitting model (Table 3) included database size and percentage of environmental BioSamples with a McFadden’s R squared value of 0.939, which indicates a good prediction accuracy. Due to multicollinearity when adding more factors into the model, we selected the two-factor model with an interaction term as the final prediction model.

**Table 3:** Multiple logistic regression estimates

	**Estimate**	**Std. Error**	**p value**
(Intercept)	− 3.90E + 00	2.38E−02	< 0.001
Number of total BioSamples	− 1.09E−05	1.62E−07	< 0.001
Percentage of environmental BioSamples	3.72E + 00	5.44E−02	< 0.001
Number of total BioSamples: percentage of environmental BioSamples	7.14E-05	6.48E−07	< 0.001

**Fig. 3:**
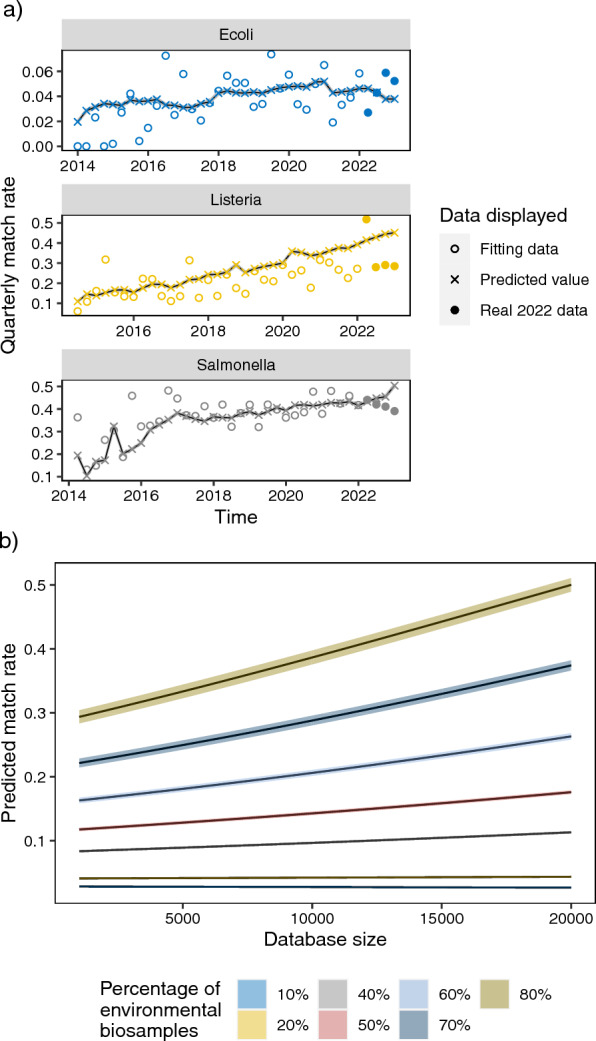
**a** Utilizing logistic regression for quarterly match rate prediction, with corresponding confidence intervals shaded in grey. **b** Employing logistic regression models to forecast hypothetical database performance across varying percentages of environmental BioSamples. Please note the distinct scales on the y-axis. Shaded areas represent confidence intervals of predicted values

Positive coefficients for the environmental percentage factor and interaction term indicated that with the same database size a higher percentage of environmental BioSamples is associated with higher match rate. Regarding the relationship between database size and the match rate, when environmental percentage is fixed and higher than 15%, larger database size is correlated with higher match rate (Fig. 3). For instance, under hypothetical conditions, when environmental percentage reaches 70%, the odds of getting matched clinical BioSamples was predicted to rise 4% with every 1000 isolates deposited into database(Fig. 3b). However, if the environmental percentage is lower than 15% and fixed, with larger database size, the tendency of match rate decreases.

To evaluate our prediction model, which was built upon data before December 31, 2021, we compared predicted match rates with actual quarterly rates in 2022. We found that the average absolute difference between predicted match rate and the actual match rate are 5% and 1% for *Salmonella* and *E. coli* respectively. The model was not as good for *L. monocytogenes* where the average absolute difference is 14%, due to the big jump of actual match rate in the first quarter.

## Discussion

The results presented here show that for the database sizes we investigated the match rate of clinical isolates to non-clinical isolates is 33% for *L. monocytogenes*, 46% for *Salmonella*, and 7% for *E. coli.* While comparisons to other studies are difficult given the estimate of a match rate is highly dependent on the composition and size of the database and the genetic threshold (SNP distance) at which a match is defined, our results are in line with what has been seen by others. For example, Sanaa et al. [9] based on NCBI Pathogen Detection data from 2018 and a SNP distance threshold of 20 (the same value used here) found the probability that a new clinical would match an existing food or environmental isolate was relatively low ~ 30% for Salmonella and ~ 12% for *L. monocytogenes*. Although lower than the values we observed, the authors note that the probability of a match appeared to be increasing.

In modeling the match rate, we found that variation exists overtime within and among foodborne pathogens in the epidemiologically informative match rate. The drastic variation is likely the primary reason that prospective modeling to estimate the probability that any future clinical sample will be a match to a non-clinical isolate is currently difficult. Although studies have found there is a seasonality to the prevalence of certain *Salmonella* serovars [10], our tests of models incorporating seasonality showed no consistent relationship, which is also likely due to the variation and erratic pattern to the match rate overtime. Perhaps this is a surprising result where even after 10 plus years of populating such databases and 750,000 isolates, as is the case with *Salmonella*, the information content and probability of a match have not stabilized. However, modeling the match rate had good performance and provides a means for estimating whether future clinical samples will match non-clinical samples in the database. Such databases will continue to routinely provide actionable information where they are a critical tool for foodborne disease surveillance and outbreak detection and resolution.

## Limitations

Limitations are discussed throughout and include, but are not limited to, the data that we analyzed do not represent a random sample and results will vary depending on the SNP threshold used to determine a match.

### Supplementary Information

Additional file 1.

## Data Availability

The data described in this Research Note can be freely and openly accessed at https://ftp.ncbi.nlm.nih.gov/pathogen/Results/Salmonella/, https://ftp.ncbi.nlm.nih.gov/pathogen/Results/Listeria/, https://ftp.ncbi.nlm.nih.gov/pathogen/Results/Cronobacter/, and https://ftp.ncbi.nlm.nih.gov/pathogen/Results/Escherichia_coli_Shigella/.
